# Biomechanics of surgical knot security: a systematic review

**DOI:** 10.1097/JS9.0000000000000298

**Published:** 2023-03-24

**Authors:** Yoke-Rung Wong, Duncan A. McGrouther

**Affiliations:** a Biomechanics Laboratory; b Department of Hand Surgery and Reconstructive Microsurgery, Singapore General Hospital, Academia, Singapore

**Keywords:** biomechanical testing, suture materials, reef knot, surgeon’s knot, racking hitch knot

## Abstract

This review aims to identify publications on quantitative biomechanical testing of surgical knot security and the physical factors that determine knot security and failure.

**Materials and Methods:**

An electronic literature search was performed in accordance with PRISMA guidelines in January 2022 utilizing the PubMed and Google Scholar databases to look for objective biomechanical studies on knot security in surgery using the primary terms ‘knot security’ and ‘biomechanical testing’.

**Results:**

Thirty-six articles were included. Twenty-four configurations of surface, laparoscopic, and arthroscopic knots were studied. Biomechanical tensile testing was used to evaluate knot security *in vitro*. Load to failure (N) and elongation at knot failure (mm) were quantified by static and cyclic testing to evaluate the knot holding capacity and failure mechanism of slippage or rupture.

**Conclusion:**

This review reassures that the knot configuration, suture materials, suture sizes, and number of throws are key factors in determining the knot’s security. Knot configuration has to be simple for laparoscopic and arthroscopic knots due to the confined space of the operating site. With the advent of stronger suture materials for high-tension surgical reconstructive procedures, there is an unmet need to understand the physical behavior of the knot and the factors that determine its resistance to slippage or rupture.

**Level of Evidence:** Level IV.

HIGHLIGHTS

- Review and provide an overview of publications on quantitative biomechanical testing of surgical knot security and the physical factors that determine knot security and failure.
- Thirty-six out of 83 studies were selected based on the literature search and inclusion/exclusion criteria.
- The knot configuration, suture materials, suture sizes, and number of throws are key factors to determine the knot security and understand the physical behavior of the knot with the advent of stronger suture materials for high-tension surgical reconstructive procedures.

## Introduction

Knots are ubiquitous and ancient for the joining of all manner of longitudinally oriented materials, and the formation of knots must surely have paralleled the use of sutures in surgery, which is an ancient art apparent in Egyptian mummies dating from 3000BC^1^.

The knots that are in everyday use in surgery are derived from traditional practice rather than experimental analysis of the physical factors that determine knot security. This review focuses on articles that have quantitated through laboratory experimentation the ability of knots to remain secure under tensional load. The mechanisms of failure by slippage or suture material rupture will be analyzed.

Knots work by achieving a static contact between two strands of suture material of various types^2^. The strands may be separate (called working ends in knot parlance), or the contact area between two regions may be achieved by folding a single length of material as in the Aberdeen knot. Essentially, the static contact is generally considered to be due to friction between the strands, and many components contribute to the frictional load, including surface roughness at macroscopic or microscopic dimensions and the area of contact, which relies on the deformability of the suture but more particularly on the knot configuration.

The ability to maintain a tight knot is defined by several physical factors, including the number of loops, often called ‘throws’ in surgical practice. For surface knots, a first throw is generally locked by the addition of a second, and further throws may be added. Endoscopic knots have a variety of different configurations determined by the anatomical space available. The tying of a surgical knot is a critical skill for surgeons to ensure knot security, which is crucial to maintaining the integrity of a tied suture. Failure of a surgical knot may result in wound dehiscence or tissue repair rupture.

This review aims to identify publications on quantitative biomechanical testing in relation to knot slippage or rupture. We reckon that the discussion and suggestions could help the surgeons gain a better understanding of the design of knot techniques and facilitate the translation of experimental results from biomechanical testing to the clinical setting.

## Materials and Methods

This systematic review was done in accordance with PRISMA guidelines^3^. An electronic literature search was performed in January 2022 utilizing the PubMed, Google Scholar, and other resources databases to look for objective biomechanical studies on knot security in surgery. The terms ‘knot security’ and ‘biomechanical testing’ were used as primary keywords for database searches. Typical patterns of knots were identified, such as those used on a visible surface or tissue layer or those used in arthroscopic procedures. Knot failure, including knot slippage or rupture, was considered. Other mechanisms of failure are excluded from this review such as stretching of the suture material or tearing out of the tissues even when the suture loop remains intact. ‘Qualitative testing’, ‘other types of testing’, and ‘case report’ were also excluded, and only English-language papers were included. The search results were combined after duplicates were removed and screened based on title and abstract screening. A full-text review of selected articles was undertaken by the two authors. Uncertainty regarding inclusion was resolved by the decision of the clinical author (Duncan A. McGrouther). The physical factors of knot security were defined, and relevant data was extracted from the articles, including figures and tables, by a single author (Yoke-Rung Wong).

## Results

### Literature search

**Figure 1:**
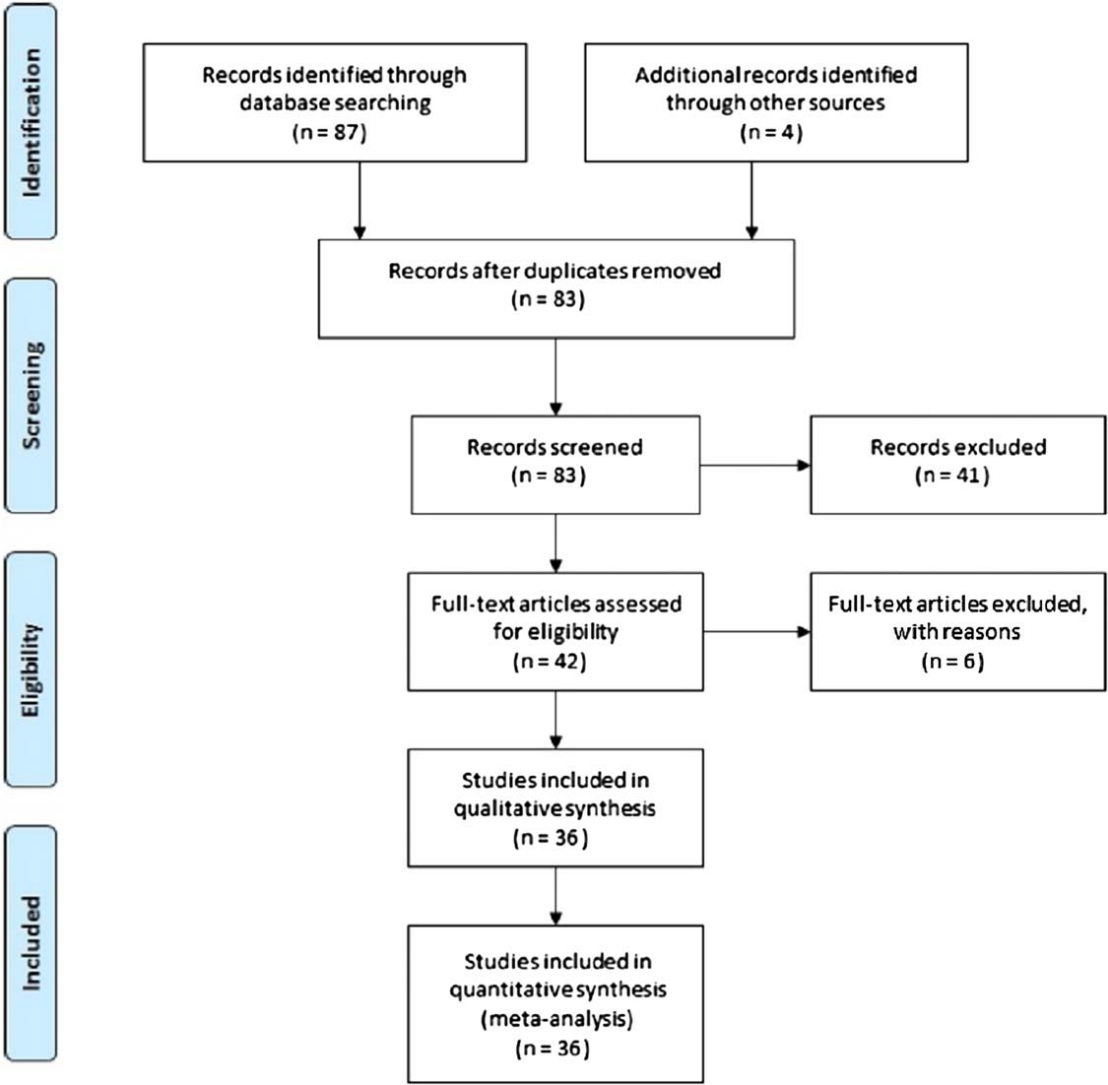
PRISMA flowchart of study progression.

As shown in Figure 1, a limited number of reports were found with objective biomechanical testing covering a range of different applications of surface and arthroscopic knots. Eighty-three articles were found to be of potential interest. After reviewing the title and abstract, 36 articles with objective data were included in this review^4^–^39^. Six articles were excluded because of other factors such as knotless technique, heat, and instrument effect. Table 1 summarizes the knot technique, suture material and size, number of throw(s), and biomechanical testing results for each identified publication. Tables 2 and 3 illustrate the surface, laparoscopic, and arthroscopic knots that were tested in the publications.

**Table 1:** Physical factors and biomechanical testing of knot security.

No	References	Knot Technique(s)	Suture Materials (Size)	Knot Configuration/Throw(s)	Biomechanical Testing	Knot, Suture Material, Maximum/Minimum Load to Failure (N) (Throw)	Suture Breakage or Knot Slippage (Rate, %)	Knot, Suture Material, Maximum/Minimum Elongation (mm) (Throw)
1	Hong *et al*.^4^	Roeder, Western, Samsung medical center (SMC), Tennessee, Surgeon	FiberWire (#2), 1.3 mm SutureTape (–)	Six throws for surgeon, 3 reversing half hitches on alternating posts (RHAPs)	Knots were tied on metal post and then soaked in saline solution for 1 min Static testing	Surgeon, SutureTape, 300.2; Western, FiberWire, 134.9	–	–
2	Ergün *et al*.^5^	Surgeon	Polyethylene UHMWPE (2-0)	Two throws	Knots were tied on 2 parallel metal rods in 3.0 mm diameter. Loop circumference lengths: 19.4 mm, 23.4 mm, 27.4 mm, 31.4 mm Cyclic testing	–	–	19.4 mm, 0.732; 31.44 mm, 1.292
3	Teo *et al*.^6^	Duncan, HU, SMC, Pretzel, Nicky’s, Square, UM	Hi-Fi suture (#2)	Four half hitches	knot was created around a metal hook. The knot was tied outside of the 8.4 mm cannula and pushed down using a single-hole knot pusher Cyclic testing, Static testing	Nicky’s, 271.3; HU, 218.9	Suture breakage (%) UM, 60	UM, 0.41; SMC, 0.67
4	Leuam *et al*.^7^	Nice, modified nice, double-twist, double-barrel, square knot of DSLS, square knot of single-stranded suture	Fiberwire (2-0)	–	Knots were tied in a loop and held by a pair of rods Cyclic testing, Static testing	Nice, 221.3; square knot of single-stranded suture, 106.6	Suture breakage (%) modified nice, 100 square knot of DSLS, 67	Nice, 1.1; Square knot of single-stranded suture, 2.03
5	McGlinchey *et al*.^8^	Forwarder, surgeon	Polyglactin 910 (#3)	2,3,4 throws for Forwarder, 5,6,7,8 throws for Surgeon	Knots were tied to the bar held by the bottom gripper Static testing	Forwarder, 163.8 (3); surgeon, 86.9 (5)	Suture breakage all knots	–
6	Westberg *et al*.^9^	Surgeon, nice, modified nice	FiberWire (#2), Ultrabraid (#5), Ethibond (#5)	Two throws for surgeon, 3 RHAPs, other knots	Knots were tied in a loop and held by a pair of rods Cyclic testing, Static testing	Surgeon, FiberWire, 256; modified nice, ultra braid, 83.2	–	Modified Nice, Ethibond (cyclic displacement/time, 1.8×10^−4 ^mm/s)
7	Corey *et al*.^10^	Wiese	FiberWire, Orthocord, UltraBraid, Ethibond (#2 for all sutures)	3 RHAPs	Knots were tied on metal dowel Static testing	Ultrabraid, 274.9; Ethibond, 142.4	Knot slippage (%) All knots, 80	–
8	Meyer *et al*.^11^	12 type of knots	Fiberwire (2-0)	–	Static testing	Cow hitch (Larks head), 224.2; Nice, 193.3	Suture breakage All knots	–
9	Chong *et al*.^12^	Arthroscopic knot	Force Fiber, FiberWire, Orthocord, Ultrabraid (#2 for all sutures)	3 RHAPs	Knots were tied on 30 mm circumferential rod Static testing	Ultrabraid, 22.8; Orthocord, 9.9	–	–
10	Jiang *et al*.^13^	Squared surgeon, Z knot, antislip knot	Fiberwire (4-0)	4=4=1, 2=2=2=2, 1=1=1=1=1, 2=1=1=1=1,	Knots were tied in a loop and held by a pair of rods Static testing	Antislip, 76.8; Z knot, 25	–	–
11	Gillen *et al*.^14^	Aberdeen, surgeon, square	Polyglactin 910 (#2, #3), polydioxanone (#2)	3,4,5,6,7,8	Knots were tied to the bar held by the bottom gripper Static testing	Aberdeen, polyglactin #3, 125 (6); square, polyglactin #2, 20 (4)	–	–
12	Hill *et al*.^15^	Nice, Surgeon, Tennessee	FiberWire, Ultrabraid, Hi-Fi, Force Fiber	1, 2 and 3 alternating post half hitches (APHHs) for Nice, 4 APHHs for Surgeon and Tennessee	Tied around a fixed diameter cyclic testing, Static testing	Nice 3 APHHs, FiberWire, 228; Nice 1 APHHs, Ultrabraid, 99	Suture breakage (%) Nice 3 APHHs, 82 Force Fiber, 64	Nice 3 APHHs, FiberWire, 0.4; Surgeon, Ultrabraid, 3.2
13	Kuptniratsai kul *et al*.^16^	Weston, Tennessee, SMC, Chula	MagnumWire, Hi-Fi, FiberWire (#2 for all sutures)	–	Knots were tied on 30 mm circumferential plastic rod Static testing	Weston, Hi-Fi, 58.8; Tennessee, FiberWire, 21.9	Knot slippage All knots	–
14	Regier *et al*.^17^	Square, Aberdeen	Polyglyconate (4-0)	–	Knots were tied as they would be in a clinical setting on cadaveric skin Static testing	Aberdeen, 152.4; Square, 128.4	Suture breakage (%) All knots, 83	–
15	Kelly *et al*.^18^	Racking hitch, Weston, Square	Force Fiber (#2, #3, #4), FiberWire (#2), Ethibond Excel (#2)	–	Each knot was tied directly on the circular testing fixture Cyclic testing, Static testing	Racking hitch, force fiber #2, 428.8; square, force fiber #2, 77.4	–	Racking hitch, Ethibond Excel, 0.09; Square, Force Fiber #2, 1.1
16	Clark *et al*.^19^	Slippage-proof, modified slippage-proof, SMC, Revo	Hi-Fi suture (#2)	–	The knots were tied on the standardized cylinder Cyclic testing Static testing	SMC, 304.2; SPK, 200.6	Suture breakage All knots Suture slippage (%) Slippage-proof, 100	SMC, MSPK, 1.23; SPK, 2.46
17	Zhao *et al*.^20^	Square, two-strand over hand locking (TSOL)	FiberWire, Ethicon, Polydioxanone (3-0 for all sutures)	3, 4 throw for square, 1 throw for surgeon with 2 throw square knot, 1 throw for surgeon with 3 throw square, 5 throw for square and TSOL knot	Specimen was kept moist with a saline solution mist Static testing	TSOL, FiberWire, 45; square, polydioxanone, 15 (3)	–	–
18	Karahan *et al*.^21^	Pretzel, SMC, Giant, Dines, Nicky’s, Tennessee	Ethibond (#2)	3 RHAPs	Loops were placed around 2 metal hooks with 2.6 mm diameter Static testing, cyclic testing	Dines, 160; Nicky’s, 120	Suture breakage All knots	Nicky’s, 0.5; Tennessee, 1.5
19	Tidwell *et al*.^22^	Square	FiberWire, Surgipro, Monosof, Maxon, Polysorb (#5, #2, 0, 2-0, and 4-0 for all sutures)	3,4,5,6 throws	Loops were placed around the 2 hooks Static testing	FiberWire #5, 306 (6); Surgipro 4-0, 10.5 (6)	Suture breakage All knots	–
20	McDonald *et al*.^23^	Surgeon	Fiberwire, Supramid, Ethibond, Multifilament stainless steel (3-0/4-0 for all sutures)	3 and 5 throws	Knots were wrapped around a spool and held with a clamp Static testing	Multifilament stainless steel 3-0, 121 (3); Fiberwire 4-0, 18.4 (3)	–	–
21	Pietschmann *et al*.^24^	Square, Revo, Fisherman, Roeder	FiberWire, Orthocord, Herculine, Ethibond Excel, PDS II (#2 for all sutures)	4 RHAPs	Knots were wrapped around a rod and then held with a clamp Cyclic testing	–	Knot slippage in dry condition (15%); Knot slippage in wet condition (7%)	–
22	Punjabi *et al*.^25^	Tennessee, Roeder, SMC, Duncan, Weston, Nicky	FiberWire (#2)	3 RHAPs	Knot was tied on a 3-mm-diameter post The free ends of the suture were rolled circumferentially from opposite directions (5 loops each) onto another 3-mm-diameter post-Quasi static testing	Load at relaxation mode (N), Elongation in percentage at relaxation mode (%)	–	–
23	Dahl *et al*.^26^	Dines, Nicky, Field, Tennessee, Snyder, San Diego, Hu, Tuckahoe, Triad	FiberWire	3 RHAPs	Loop were tied around a 1.5 cm diameter pulley Static testing	Tennessee, 269; Synder, 69	–	–
24	Barber *et al*.^27^	Weston, Tennessee, Duncan, SMC, Revo, San Diego	Ethibond, FiberWire, Orthocord, Hi-Fi, Ultrabraid, Force Fiber, MagnumWire, MaxBraid PE (#2 for all sutures)	4 RHAPs	Knots were tied around a standardized, 6.5-cm diameter cylinder Cyclic testing, Static testing	Revo, 281; Duncan, 150; FiberWire, 260; Ethibond, 144	Suture breakage (%) SMC, 98.75 Suture breakage (%) Ethibond, 77.6	–
25	Nishimura *et al*.^28^	Antislip, Reef	Ethibond, Fiberwire, Nespron	3,4, 5, 6 throws for antislip, 3, 4, 5, 6, 7, 8. and 9 for reef	A loop of suture material with a knot approximately 50 mm in length was set on an S-shaped hook (6 mm in diameter) Static testing	Antislip, FiberWire, 590 (6); Reef, Ethibond, 80 (3)	Suture breakage (%) Antislip, Ethibond, Knot slippage (%) Reef, Nespron and FiberWire, 100 (3,4)	–
26	Elkousy *et al*.^29^	Open and square knots, Duncan, Open and revo	Ethibond (#2), FiberWire (#2)	Six throws for open and square, 3 RHAPs, Duncan and revo	Knots were tied through an 8-mm cannula with use of an arthroscopic knot pusher Suture was soaked in saline between 30 seconds and 2 minutes before knots were tied Cyclic testing, Static testing	Open and square, FiberWire, 264; Open and revo, Ethibond, 142	Suture breakage (%) Ethibond, 100 Knot slippage (%) FiberWire, 75	Open and square, Ethibond and FiberWire, 0.15; Open and revo, FiberWire, 0.3
27	Hassinger *et al*.^30^	Dines, Duncan, Field, Giant, Lieurance Modified Roeder (LMR), Nicky’s, SMC, Snyder, Tennessee, Weston	Ethibond (#2)	3 RHAPs	Knots were tied on 2 pulleys Static testing	Dines, 149; Duncan, 106	–	–
28	Jianmongkol *et al.* 31	Surgeon, Square	Nylon monofilament sutures (4-0)	2 throws	Static testing	Only stiffness was presented	Suture breakage (%) Surgeon, 47.22 Knot slippage (%) Square, 58.33	–
29	Elkousy *et al*.^32^	Weston, Square, Duncan, Nicky’s	Surgidac (#2)	Three RHAPs for Duncan and Nicky’s, 1 half-hitch for Weston, 3 RHAPs for Weston, 6 throws for square	Knots were tied in between 2 metal rings Static testing, Cyclic testing	Square, 178; 1 half-hitch for Weston, 129	Suture breakage (%) Square, 100 Knot slippage (%) 1 half-hitch for Weston, 83	1 half-hitch for Weston, 0.1; Duncan, 0.3
30	Kim *et al*.^33^	Duncan, Field, Giant, SMC	Ethibond (#2)	0, 1, 2, 3, 4, 5 RHAPs	knots were tied around a metal bar, 5 mm in diameter Static testing	Giant, 5 RHAPs, 143; Duncan, Field and SMC, 0 RHAPs, 0	Suture breakage (%) SMC, Giant and Field, 4,5 RHAPs, 100 Knot slippage (%) Giant, 1 RHAPs, 100	–
31	Lo *et al* .34	Duncan, Nicky’s, Tennessee, Roeder, SMC, Weston, Surgeon	Ethibond (#2), FiberWire (#2)	Without RHAPs, With 3 RHAPs	knots were tied over a plastic post to create a 30-mm suture loop Static testing	Surgeon, FiberWire, 3 RHAPs, 197; Tennessee, Ethibond, 10	Knot slippage All knots	–
32	Babetty *et al*.^35^	Square and surgeon (alternating sliding knots)	Silk, Nylon (2-0 and 4-0 for all sutures)	S//S//S//S, S#S#S#S, S=S//S=S, SXS#SXS	Knots were tied around 2 cylindrical woodened rods Static testing	S=S//S=S, Silk, 23 (2-0); S#S#S#S, Nylon (4-0)	Suture breakage (%) S=S//S=S, Silk, and SXS#SXS, Nylon, 100 (2-0 and 4-0); Knot slippage (%) S=S//S=S, Nylon, 60 (4-0)	–
33	Rodeheaver *et al*.^36^	Granny	Lactomer, Polyglactin 910 (3-0 for all sutures)	2, 3, 4, 5 throws	Knots were tied around the plastic mandrel (3.0 cm diameter) Static testing	Lactomer, 37.2 (3); Polyglactin 910, 1.7 (2)	Suture breakage (%) Lactomer, 100% (2) Knot slippage (%) Polyglactin 910, 100 (2)	–
34	Mishra *et al.* 37	Overhand, Duncan, Roeder, Snyder, Square	Maxon, Ticron (#1 for all sutures)	3RHAPs, 4 throws for square	Knots were tied around two rings placed in the testing chamber filled with normal saline Static testing, cyclic testing	Square, Maxon, 178; Overhand, Ticron, 57.2	Suture breakage (%) Duncan, Roeder, Snyder, Square, 90, Knot slippage (%) Overhand, 100	Roeder, Snyder, Square, Ticron, 0.1; Duncan, Overhand, Maxon, 11
35	Dang *et al.* 38	Square, Granny, Surgeon	Goretex, Prolene (2-0 for all sutures)	3, 5, 7 throws	Knots were mechanically tied at a constant rate of loading with predetermined tension Static testing	Surgeon, Goretex, 25.6 (3); Surgeon, Prolene, 12.5 (7)	–	–
36	Rosin *et al.* 39	Square, Granny, Surgeon	Ethicon, Dexon Plus, Vicrvl, PDS, Prolene, Ethlcon (#2 for all sutures)	3, 4, 5, 6, 7 throws	Knots were tied around a 4 cm diameter cylinder Static testing	–	–	–

– denotes as ‘not applicable’, ‘not specified’, or ‘not tested’.

**Table 2:**
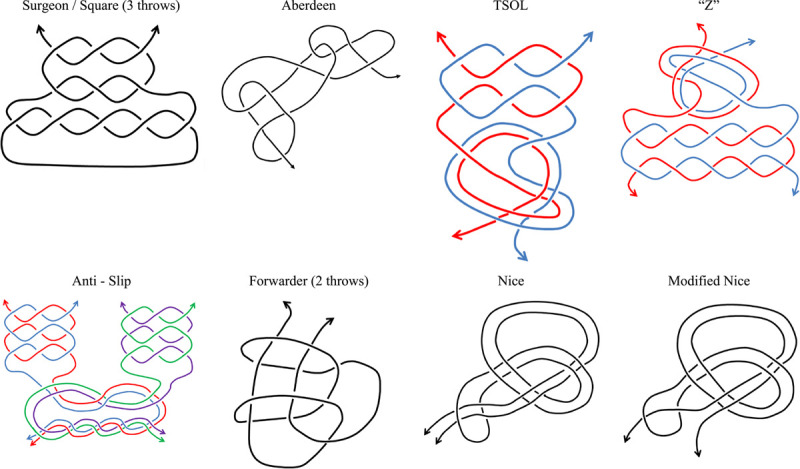
Illustration of surface knots.

**Table 3:**
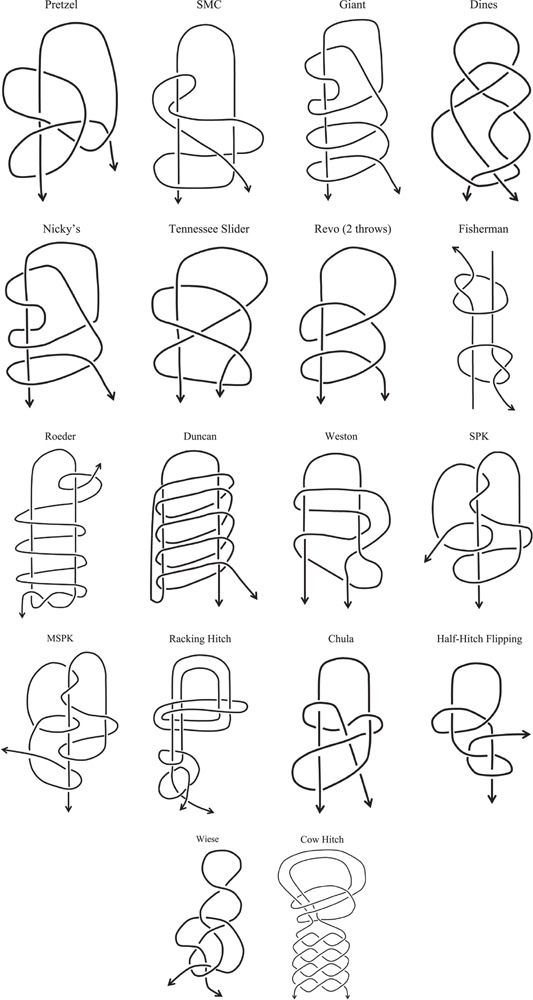
Illustration of laparoscopic and arthroscopic knots.

#### Knot techniques – configurations of surface knots

Various popular configurations were tested. For surface knots, the square knot (reef knot) and the surgeon’s knot, which has a double wrap-around on the first throw, were the most studied. This double loop is formed by one strand of suture spiraling around the other, generally achieved by wrapping the suture strand twice around a surgical instrument before grasping the other ‘working end’. Although generally believed to be secure in clinical practice, both reef and surgical knots were noted to allow slippage at high tensile loading. This was a particular problem for strong but stiff suture materials. McDonald *et al*.^23^ and Jiang *et al*.^13^ required five throws when tying Fiberwire 4-0 in order to achieve up to 76.8 N of load to failure but Tidwell *et al*.^22^ recommended an additional throw to enhance the load to failure to 306 N. Adopting a different approach, Zhao *et al*.^20^ described a new knot for high-tension requirements, the two-strand overhand locking, which holds the two strands of suture material in contact over a long length, resulting in a larger frictional surface to resist slippage. However, only 45 N of load to failure was reported in their study using Fiberwire (five throws). Regier *et al*.^17^ investigated the use of different suture materials to make an Aberdeen knot for skin closure in cadaver animal skin and were able to gain 152.4 N using polyglyconate 4-0. They emphasized the benefit of the smaller size of this knot configuration, which is formed on a single strand of material looped upon itself. Gillen *et al*.^14^ compared Aberdeen knots with square and surgeons’ knots (20 N; Polyglactin #2; four throws) and noted the greater strength of the Aberdeen knots (125 N; Polyglactin #3; six throws) and their capacity to reduce slippage. McGlinchey *et al*.^8^ noted suture breakage issues in veterinary practice in the use of strong suture materials with a forwarder knot (163.8 N; Polyglactin #3; three throws) in which one working end wraps around the other, giving a long length of contact between the two working ends. Westberg *et al*.^9^ compared strong polymer suture materials (Fiberwire, Ultrabraid, and Ethibond) and found that these sutures were stronger than wire sutures for cerclage bone fracture fixation, but their laboratory measurement configuration of two loops or throws only makes analysis of slippage or breaking strain data difficult.

#### Knot techniques – configurations of laparoscopic and arthroscopic knots

Endoscopic and arthroscopic knots are applied at different body sites, and the general requirement in knot tying is to allow slippage of individual loops as the knot is being formed but no slippage when the knot is complete. The knots are built up loop by loop, and advanced one loop at a time through a cannula introduced through a minimally invasive incision. Each loop is advanced into the wound by means of a knot pusher and tightened once in position by direct pressure of the pusher or by traction with the pusher pulling the suture distal to the forming knot (past pointing). A variety of ingenious knots have been described using these techniques, and the specific knot configuration is generally locked in place by a series of half hitches.

Arthroscopic knots have complex configurations (Table 2). Karahan *et al* .21 endeavored to describe a simpler configuration requiring fewer individual manoeuvres. They reported the highest load to failure (160 N) for the Dines knot using Ethibond #2. Pietschmann *et al*.^24^ demonstrated that the knots behaved differently in wet or dry environments. Punjabi *et al* .25 compared several configurations of knots and found that mechanical characteristics such as stress relaxation were more dependent on stretching of the suture material than slippage of the knot. Clark *et al*.^19^ compared a hypothetical ‘slippage-proof knot’ with other configurations in general use but found that the widely used Samsung Medical Centre knot had the highest load to failure (304.2 N) using Hi-Fi #2. Kelly *et al* .18 demonstrated increased strength by having additional half hitches for the racking hitch knot (428.8 N; Force Fiber #2). However, Kuptinatsaikul *et al.* 16 noted several standard knots including Samsung Medical Centre all failed by slippage at loads of less than 50 N. Corey *et al*.^10^ reported knot slippage at more than 274 N but the same knot configuration using the same suture material (Ultrabraid) was found to be less than 23 N by Chong *et al*.^12^. They noted a tendency for the knot to ‘flip’ under load, changing its three-dimensional shape and mechanical characteristics. Meyer *et al* .11 described better load bearing with a double-stranded suture for Cow hitch knot (224.2 N; Fiberwire 2-0) after comparing with 11 types of other knots. Leuam *et al*.^7^ showed in a variety of double-stranded loop suture knots that square knots were more likely to fail by slippage for single-stranded suture (106.6 N) than more complex configurations such as Nice knots (221.3 N) using Fiberwire 2-0.

## Discussion

The incidence of knot failure in clinical practice is not known, but it is a common clinical observation that monofilament square or surgeon’s knots can untie, particularly if wet or subject to detergent cleaning with lowered surface tension. An important factor is how tightly the knot has been tied, which is difficult to standardize in laboratory testing or clinical practice. The studies included here illustrated knot slippage of square and surgeons’ knots at high tension, typically 80–100 N although results in laboratory testing may not be achieved in the living patient. The need for high tensional strength is only required in certain operations, such as tendon repair or hernia repair, and much lower tensile strain is satisfactory in routine wound closure. Suture slippage or unraveling is however possible in a wet and mobile environment.

For all surgical knots, we are aware that the advancement of high-strength suture material has taken a paradigm shift from monofilament nylon (Prolene) or polypropylene (Supramid) to high molecular weight, long chain, multistrand, braided polyester (Fiberwire and Orthocord). In general, the monofilament suture has a single strand with smooth surface while the braided polyester type suture has bundles of strands which are woven in a unique pattern. In order to achieve a high load to failure, stronger suture materials and a larger suture size (minimum #2) are used in some applications. Among all the studies in this review, racking hitch knot with force fiber (#2) could achieve the highest load to failure (428.8 N).

This review shows that stronger suture materials present difficulty in forming knots that resist slippage. The use of five or six loops to counter this could create a bulky knot that causes excessive tissue reaction and foreign body reaction to the sutures, possibly leading to wound breakdown. Up to eight throws for the Aberdeen, Surgeon’s, and Square knots were tested by Gillen *et al* .14. They found that an Aberdeen knot with six throws using polyglactin (#3) could achieve 125 N of load to failure. However, Regier *et al*.^17^ reported that Aberdeen knot with four throws using polyglyconate (4-0) could achieve a higher load to failure (152.4 N). Therefore, it is difficult to define the optimal suture size and configuration for a particular knot, in this case the Aberdeen knot, because it depends on the combination of suture material and suture size, not to mention other factors such as the number of throws.

The formation of surgical knots with different numbers of throws is particularly challenging for high-strength suture material in terms of suture size, flexibility, surface configuration, and roughness, let alone considering the variables of whether the suture is wet or dry. Although biomechanical studies have shown that the braided polyester suture has superior mechanical strength, we have not found any testing data to evaluate the friction of the braided polyester suture. In the general testing of friction between two objects, friction increases as normal load increases. The fundamental intuitive concept of tying a knot with an increasing number of throws is that higher frictional force can be obtained as a result of greater contact area between loops of suture. Zhao *et al* .20 found that the coefficient of friction of suture material directly affects the knot holding strength. Increasing the number of throws for braided polyblend suture helps to prevent knot loosening, which seems to occur from ‘plastic memory’. We advocate that there is a need to gain a better understanding of the way in which the combination of mechanical strength and friction of suture materials affects knot security with respect to different suture materials.

In this review, we also found that suture size#2, was frequently used for laparoscopic and arthroscopic knots, but more sizes of #3, #4 or 3-0 and 4-0 are in common use for surface knots. Larger suture sizes may contribute to higher repair strength but not to knot security. In fact, higher repair strength usually demands a higher knot holding capacity which may fail by causing the knot to unravel rather than suture breakage taking place. Therefore, the suture size may not be the only factor to consider from the perspective of biomechanical performance when considering the surgical procedure.

A surgical suture consists of two components: the suture and the knot. Higher load at the knot is expected since the strong mechanical suture can tolerate higher loading. Increasing the number of throws is the clinical option to increase the contact surface of suture for higher frictional force and thus prevent knot unraveling due to slippage. On the other hand, other factors such as increasing the normal load on the contact surface, possibly by changing the knot configuration, could also increase the frictional force. We have not found any study that investigates the configuration of a knot with respect to the friction coefficient of suture material or normal load.

Another finding also demonstrates that cyclic testing should be adopted to test knot security for different suture materials and knot techniques because the cyclic loading applied to the specimens simulates the actual tissue loading and unloading during rehabilitation. However, the testing method should be standardized according to the actual protocol of rehabilitation so that the experimental results are translatable to the clinical setting. Although testing methods could be optimized according to the rehabilitation protocols, and the physiology and anatomy of the human body, the in-vitro models have limitations in reflecting the in-vivo information on the interaction between the knot and the surrounding tissue during the healing process.

We are also aware of utilizing the finite element analysis to investigate the knot security. The finite element analysis is a numerical method that solves a set of partial differential equations simultaneously in two- or three-dimensional models under different physical conditions. For example, Qwam Alden *et al* .40 reported a finite element model of the single throw of a surgical knot on a single strand using fishing line (monofilament nylon). It was found that the force required to break knotted fishing line was ~50% lower than the untied fishing line due to the stresses from bending, twisting, and frictional contact. Chow *et al*.^41^ noted that the slippage force of three throws (5.57±1.17 N) was higher than that of two throws (1.85±0.93 N) for surgical knots made of Polyglactin 910 (Vicryl; Ethicon Inc., Somerville, NJ), suggesting that three throws were more resistant to slippage despite the additional time required to construct them. Although we are mindful of the potential application of finite element analysis to investigate the surgical knot’s security, it is believed that the inclusion of finite element analysis provides very little information and has a low impact on our review paper. Therefore, we have excluded the finite element analysis from this review.

## Conclusions

In conclusion, the knot configuration, suture materials, suture size, and number of throws are key factors in determining the knot security. Knot configuration has to be simple for laparoscopic and arthroscopic knots due to the confined space of the operating site. With the advent of stronger suture materials for high-tension surgical reconstructive procedures, there is an unmet need to understand the physical behavior of the knot and the factors that determine its resistance to slippage or rupture.

## Ethical approval

NA.

## Sources of funding

This work was supported by the Surgery Academic Clinical Program grant (Biomechanics Lab Programme), and the Singapore Ministry of Health’s National Medical Research Council under its NMRC/CG1/007/2022-SGH.

## Author contribution

All authors contribute to the following:

1. Performing the conception and design of the study, publication selection, interpretation of data.
2. Drafting the article.
3. Final approval of the version to be submitted.

## Conflicts of interest disclosure

None.

## Research registration unique identifying number (UIN)

1. Name of the registry: NA.
2. Unique identifying number or registration ID: NA.
3. Hyperlink to your specific registration (must be publicly accessible and will be checked): NA.

## Guarantor

Professor Duncan Angus McGrouther.
